# A review of dengue diagnostics and implications for surveillance and control

**DOI:** 10.1093/trstmh/trz068

**Published:** 2019-07-31

**Authors:** Nader Raafat, Stuart D Blacksell, Richard J Maude

**Affiliations:** Mahidol Oxford Tropical Medicine Research Unit, Faculty of Tropical Medicine, Mahidol University, 420/6 Rajvithi Road, Rajthevee, Bangkok, Thailand; Mahidol Oxford Tropical Medicine Research Unit, Faculty of Tropical Medicine, Mahidol University, 420/6 Rajvithi Road, Rajthevee, Bangkok, Thailand; Centre for Tropical Medicine and Global Health, Nuffield Department of Medicine, University of Oxford, Oxford, UK; Mahidol Oxford Tropical Medicine Research Unit, Faculty of Tropical Medicine, Mahidol University, 420/6 Rajvithi Road, Rajthevee, Bangkok, Thailand; Centre for Tropical Medicine and Global Health, Nuffield Department of Medicine, University of Oxford, Oxford, UK; Harvard TH Chan School of Public Health, Harvard University, Boston, MA, USA

**Keywords:** dengue, diagnosis, epidemiology, surveillance

## Abstract

Dengue is the world’s most common arboviral infection, with almost 4 billion people estimated to be living at risk of dengue infection. A recently introduced vaccine is currently recommended only for seropositive individuals in a restricted age range determined by transmission intensity. With no effective dengue vaccine for the general population or any antiviral therapy, dengue control continues to rely heavily on vector control measures. Early and accurate diagnosis is important for guiding appropriate management and for disease surveillance to guide prompt dengue control interventions. However, major uncertainties exist in dengue diagnosis and this has important implications for all three. Dengue can be diagnosed clinically against predefined lists of signs and symptoms and by detection of dengue-specific antibodies, non-structural 1 antigen or viral RNA by reverse transcriptase–polymerase chain reaction. All of these methods have their limitations. This review aims to describe and quantify the advantages, uncertainties and variability of the various diagnostic methods used for dengue and discuss their implications and applications for dengue surveillance and control.

## Introduction

Dengue virus (DENV) is the most common arbovirus worldwide, with >128 countries showing evidence of endemic dengue transmission and almost 4 billion people living in areas at risk of dengue infection.^1^ In 2013, the Global Burden of Disease Study showed that dengue incidence has more than doubled every decade from 1990 to 2013, unlike other communicable diseases.^2^ The most commonly cited figure, including by the World Health Organization (WHO),^3^ is an estimated 96 million symptomatic infections per year (95% credible interval 67–136 million).^3,4^ However, the high asymptomatic rate means the actual burden is likely to be much higher, with an estimated 390 million total annual infections.^4^ There is no specific antiviral treatment for dengue, so supportive management requires early recognition. A vaccine has recently been introduced, but due to differential efficacy and safety issues in seronegative individuals, its use is restricted to people who have serological evidence of previous infection and to age groups at highest risk of severe disease, most typically ages 9–45 y.^5^ Therefore dengue control still relies primarily on vector control measures, including removal of breeding sites and fogging with insecticides.

Dengue can be diagnosed clinically and confirmed by a variety of methods, including anti-DENV antibodies, non-structural protein 1 (NS1) antigen or DENV-specific nucleic acid detection. Confirmation of dengue diagnosis is helpful in guiding supportive clinical care, particularly for atypical cases, and reducing the need for expensive investigations and treatments for alternative diagnoses. It is also important for surveillance to guide the implementation of dengue control measures. The WHO advises that dengue-specific laboratory tests are often not required for acute management of cases but should be performed to confirm the diagnosis.^6^

However, confirmatory testing is often not done. For example, according to the Pan-American Health Organisation (PAHO), only 209 178 of 561 356 (37.3%) reported dengue cases in 2018 were laboratory confirmed in the Americas. In 2013–2018, this figure ranged from 13.6% to 37.3%.^7^ Cost is likely to be a significant factor, with the rate of confirmation ranging from 0% to 100% between countries in the PAHO region, being higher in richer countries such as the USA. In resource-poor settings where dengue is prevalent, clinicians may be forced to rely on their clinical judgement, as accurate diagnostics tend to be expensive, time-consuming or both. No similar data could be obtained for the Southeast Asia and West Pacific regional offices. Nonetheless, in a meeting of Asia–Pacific dengue prevention boards, only 12 of 22 attending countries (55%) confirmed all officially reported cases with laboratory testing.^8^ It therefore seems likely that most reported cases are only clinically diagnosed, highlighting a dire need for practical and affordable dengue diagnostics that can be widely used.

Furthermore, diagnostic methods vary in their sensitivity and specificity, meaning that not all reported cases of dengue are equally accurate. As the diagnostic test used (if any) is rarely reported alongside dengue cases, it is difficult to account for diagnostic uncertainty, which varies between and within countries depending on the protocol and accessibility of different tests. As uncertainty in reported figures is one of the biggest challenges to calculating ‘true’ numbers of dengue cases,^2,4^ this review aims to describe the different methods of dengue diagnosis, their practical and diagnostic limitations and the resulting implications for dengue surveillance and control. Virus isolation will not be discussed in this review because of its prohibitive cost and long time to result (1–2 weeks),^9^ which limits its utility for guiding clinical management or large-scale surveillance, particularly in low-resource settings. The initial search strategy is described in Box 1.

Box 1. **Review search strategy**

**Table:** Initially PubMed was searched for the following strings for each section and other relevant articles were sought using article citations and the PubMed ‘Similar articles’ feature, as well as more specific search strings to answer questions that arose during the writing of the review.

**Clinical diagnosis:** (diagnosi* OR definition* OR guideline*) AND dengue[MeSH Terms] **Tourniquet test:** dengue AND tourniquet **Antibody serology:** (dengue AND (IgM OR IgG OR serolog* OR ELISA OR assay*) AND (diagnost* OR diagnosi*) AND evaluat* **Non-structural protein 1 antigen:** (dengue AND NS1 AND (diagnost* OR diagnosi*) **Nucleic acid testing:** PCR AND dengue AND evaluation AND (diagnosis OR diagnostic*) AND (sensitivity OR specificity OR accuracy) **Diagnostic evaluations:** quality AND (control OR assessment OR evaluation* OR evaluat* OR performance) AND dengue AND (lab OR laborator* OR test*) AND (diagnosis OR diagnostic*)

## Clinical diagnosis

### Clinical examination

In the 1997 WHO guidelines,^10^ dengue is classified into dengue fever (DF), dengue haemorrhagic fever (DHF) and dengue shock syndrome (DSS). DF is defined as fever with two or more of the following: headache, retro-orbital pain, myalgia, arthralgia, rash, haemorrhagic manifestations (including positive tourniquet test) or leukopenia. DHF is distinguished from DF based on haemorrhagic manifestations, thrombocytopenia and evidence of plasma leakage. A case of DSS must meet all DHF criteria and show evidence of circulatory failure.^10^ In the revised 2009 case definition, cases were classified into dengue and severe dengue (SD).^9^ Here, dengue is defined as fever plus two or more of the following: nausea/vomiting, rash, aches and pains, positive tourniquet test, leukopenia or any warning sign (outlined in the guidelines). The criteria for SD are evidence of severe plasma leakage, bleeding or organ involvement (e.g. liver aspartate transaminase/alanine aminotransferase>1000).^9^ In the 1997 guidelines, four of the criteria for DHF were patient-reported symptoms that could not be easily verbalized by young children or observed directly by the physician. This was amended in 2009 to mainly observable clinical signs with only one patient-reported symptom in the criteria for SD.^11^

Intriguingly, the WHO guidelines are not always strictly used in the clinical diagnosis of dengue. A study comparing WHO guideline performance with clinicians’ subjective diagnosis found clinician diagnosis to be more specific but less sensitive.^12^ Clinicians’ reliance on intuition and expertise could be due to the lack of WHO guideline specificity, further confounding the interpretation of reported dengue cases, particularly across different regions. In the absence of widely used laboratory confirmation, a uniform case definition is necessary to allow comparisons across regions to be made, as well as an assessment of uncertainty associated with current case definitions (the most widely used diagnostic method).

### Tourniquet test

The tourniquet test for capillary fragility is cited in both WHO guidelines as a diagnostic sign for dengue. Requiring only the use of an inflatable blood pressure cuff, it is quick and easy to perform. However, a meta-analysis of 16 studies found poor diagnostic performance, with a pooled sensitivity and specificity of 58% (95% confidence interval [CI] 43 to 71) and 71% (95% CI 60 to 80), respectively; albeit with a high level of publication bias.^13^ Similarly, a retrospective analysis of >28 000 tourniquet tests found no association between test results and final laboratory-confirmed diagnosis or dengue severity.^14^ Increasing the diagnostic cut-off from >10 petechiae to >20 petechiae did not result in the expected decrease in sensitivity and increase in specificity. This poor biological correlation between dengue infection and capillary fragility may be underlying the test’s poor diagnostic performance.^13^ Combined with practical considerations such as difficulty of interpretation in different skin colours and uncertainties around its positivity in other flavivirus infections,^14^ it may be time to forgo the tourniquet test as a diagnostic criterion for dengue.

## Antibody serology

Measuring anti-dengue immunoglobulin M (IgM) and/or IgG antibodies using an antibody-capture enzyme-linked immunosorbent assay (ELISA) is the most widely used method of confirming dengue diagnosis, because it is relatively easy to perform compared with techniques such as nucleic acid detection, although laboratory equipment and trained staff are still needed.^15,16^ However, increases in antibody titres are not immediate, meaning IgM ELISAs are <50% sensitive for at least 4 d after symptom onset in primary infection, reducing their usefulness in clinical management.^17^ Secondary cases, which are more severe, have lower IgM titres, undetectable in >20% of cases.^18^

In contrast, the IgG titre is higher in secondary infections, and therefore the IgM:IgG ratio can be used to distinguish primary from secondary infections in the acute phase of disease. A cut-off of 1.2–1.4 is used by some laboratories and commercial vendors, although this varies between laboratories.^9^ A recent study used serial blood samples from 105 primary and 144 secondary infections to determine the optimal cut-off, with a convalescent plaque reduction neutralization test (PRNT) as the reference standard.^19^ They found that varying the cut-off depending on the number of days after symptom onset outperformed static cut-off strategies. Their cut-off for defining primary infections using the IgM:IgG ratio of in-house ELISAs ranged from 1.8 at day 2 to 1.0 at day 7, giving a sensitivity, specificity and accuracy of 90%, 77% and 84%, respectively.

Furthermore, given the earlier increase and higher peak titre of IgG in secondary infections, it was possible to reliably classify infections using Panbio Indirect IgG (Alere, East Brisbane, QLD, Australia) titres alone, with a cut-off ranging from 9.3 units at day 2 to 53 units at day 7 to define a secondary infection. From day 3 onwards, this differed from the manufacturer’s cut-off of 11 units. Time-varying IgG cut-offs performed very similarly to the IgM:IgG ratio, with a sensitivity, specificity and accuracy of 91%, 78% and 85%, respectively. Although they did not validate their model in an independent testing set, an earlier study found similar results using PanBio IgG titres, with cut-offs ranging from 7.5 units at day 0 to 40 units at day 20,^20^ although clearly further research is needed to validate specific cut-offs. Overall, these results demonstrate that patient-reported days after symptom onset, while subjective, can greatly aid the interpretation of dengue antibody serology.

A major limitation in using antibody serology assays in dengue-endemic regions is antibody persistence from previous infections. For example, IgM circulates for about 60 d and IgG circulation is lifelong, meaning positive results would not distinguish recent from current dengue infections, making single samples difficult to interpret with confidence.^9,15,21^ Therefore, paired (acute and convalescent) samples are needed to detect seroconversion and confirm active dengue infection. In practice, these samples are often difficult to obtain, as patients may not return for convalescent sample collection.^9,21^

Another limitation of antibody serology is cross-reactivity with related flaviviruses, increasing uncertainty in regions where these co-circulate. For example, 8/19 (42%) sera collected after yellow fever vaccination were dengue IgM positive, despite none of them being positive before vaccination.^22^ Similarly, all commercial serology kits evaluated displayed some level of cross-reactivity with anti-Zika IgM, even when concomitant dengue infection was excluded.^23,24^ All Zika patients were also false positive for dengue IgG antibodies >14 d after symptom onset, substantially higher than the background seroprevalence of 53% in the same population.^24^ Consequently, detection of anti-DENV antibodies in a single sample during the acute phase could be the result of past infection with dengue or a related flavivirus. With increases in flavivirus vaccination rates (including dengue), transmission rates and travel to/from endemic regions, interpretation of antibody serology becomes increasingly difficult, particularly in the absence of a paired convalescent sample. Of note, no studies looking at chikungunya virus cross-reactivity were found, although this could be due to publication bias against negative results.

Even if performed correctly, antibody-capture ELISAs have good but not perfect sensitivity and specificity and these vary between different commercial kits, increasing uncertainty around reported dengue figures. Performance characteristics of commercial antibody serology kits are presented in Table 1. Overall, antibody ELISAs provide a cheap method of testing dengue infection but need to be interpreted with caution in areas of high flavivirus co-transmission/vaccination, particularly if only one sample is available, and thus are most likely to be useful in non-endemic settings.

**Table 1.** Summary of commercially available antibody serology kit diagnostic performance

Antibody	Kit	Sensitivity (%)	Specificity (%)	Reference
IgM	DENV Detect IgM Capture ELISA (InBios International, Seattle, WA, USA), catalogue number: DDMS-1	92	94	^25^
	Panbio Dengue IgM Capture ELISA (Abbott, Macquarie Park, NSW, Australia), catalogue number: E-DEN02M	89	88	^26^
	SD Bioline Dengue IgM ELISA (Standard Diagnostics, Suwon, Korea), catalogue number: 11EK20	85	97	^26^
IgG	Panbio Dengue Virus IgG Capture ELISA (Abbott, Macquarie Park, NSW, Australia), catalogue number: E-DEN02G	56	95	^26^
	SD Bioline Dengue IgG ELISA (Standard Diagnostics, Suwon, Korea), catalogue number: 11EK10	89	64	^26^

### Serostatus for vaccination

The recently licensed dengue vaccine is recommended for use in seropositive individuals 9–45 y of age,^5^ due to an increased risk of severe dengue in seronegative recipients.^27^ As a result, the WHO recommends pre-vaccination screening of an individual’s serostatus as the preferred option. Alternatively, a high population seroprevalence would mean the risk of harm to seronegative recipients is outweighed by the benefit to a large number of seropositive recipients. In both strategies, however, reliable diagnostic tests for serostatus are essential.^5^

Given the time taken for ELISA serology, it would require two visits from potential vaccine recipients, which could reduce vaccine uptake, and thus the development of reliable rapid diagnostic tests (RDTs) for serostatus screening should be a priority.^28^ As most RDTs are developed for use in acute dengue infection, they are often not sensitive enough to detect low-level antibodies from past infection. A recent systematic review of IgG RDTs demonstrated sensitivities of 75–98% and specificities of 85–100% (compared with ELISAs) in secondary/convalescent samples.^29^ Interviews with diagnostic test manufacturers indicated that increasing RDT sensitivity to more closely match ELISAs for serostatus screening should be technically feasible, a much-needed development for vaccine implementation.^29^

In addition, following dengue vaccination, high serology false positivity was observed, confounding the use of serology for surveillance/diagnosis as dengue vaccination is rolled out.^30^ Algorithms for the interpretation of dengue diagnostic tests in this context have been proposed in response, although not validated.^31^

### PRNT

The gold standard for determining previous dengue exposure (and vaccine immunogenicity) is the PRNT. This measures the serum titre needed for a specified reduction (usually 50–70%) in virus infectivity of a cultured monolayer of cells. Serotype-specific neutralising antibody titres can thus be calculated by culturing with individual serotypes.^32^ Serotyping is important for accurate modelling, as severe disease tends to be associated with secondary, heterotypic infection, therefore models predicting outbreaks will need accurate data on serotype exposure history.

The serotype specificity (unlike ELISA) and sensitivity of PRNTs outside the viraemic period (unlike PCR) allows monitoring of dengue exposure history and population seroprevalence, which is necessary for vaccination programmes. For example, a recent cross-sectional study used PRNTs to identify the effects of age and geographical location on seropositivity. The proportion of patients with multitypic neutralising antibodies increased from 28.3% in the 1–4 y age group to 63.1% in the 15–18 y age group, while the proportion of naïve subjects decreased from 4.7% to 0%. In addition, serotype-specific data identified the dominant serotype in each subregion and confirmed co-circulation of all four serotypes.^33^ Thus large-scale PRNT surveillance allows monitoring of temporal/geographical trends in the force of infection, population seroprevalence (particularly multitypic seroprevalence in adults that is indicative of infection with multiple serotypes) and serotype dominance, which would aid in tailoring public health interventions accordingly.

Although the gold standard for determining past exposure, PRNT sensitivity and specificity are not perfect for determining infecting serotype. A cohort study among 204 Thai schoolchildren found PRNTs could predict the infecting dengue serotype with an accuracy of only 67.6% (compared with PCR) when pre- and post-infection antibody titres are compared and 60.3% when only post-infection titres were used (the more likely scenario in epidemiological studies).^34^

Unfortunately, PRNTs are costly, labour intensive and not amenable to high-throughput, which has limited their use in low-resource settings and in large-scale surveillance.^32^ Furthermore, despite WHO guidance for standardization, variation in assay methodology plagues PRNTs.^32^ Factors such as cell line, viral passage and the use of complement significantly affect PRNT titres,^35^ once again highlighting the need for standardization in dengue diagnostics to allow meaningful between-lab comparisons. Novel high-throughput techniques are also under development that aim to distinguish multiple flaviviruses simultaneously and reduce the time and cost of PRNTs.^36^ Overall, as they become cheaper and faster, PRNTs are likely to play crucial roles in monitoring seroprevalence to plan vaccination programmes and monitor vaccine immunogenicity/efficacy.

## NS1

NS1 is a conserved glycoprotein that is secreted from infected cells as a hexamer and can be measured to detect dengue infection.^15^ Unlike IgM and IgG, it is present during the acute viraemic phase of infection,^37^ consistent with its postulated role in viral replication.^38^ Its early presence, specificity to dengue and abundance in sera make it useful for early diagnosis, and the need for only a single sample allows it to inform clinical management. These features make NS1 the ideal candidate for an RDT, with many commercial tests available or in development in addition to existing ELISA detection methods.^21^ RDTs can be useful in swiftly detecting (and responding to) imported cases of dengue at airports to prevent its spread across borders during epidemics^39^ and may help increase the frequency of confirmatory testing. A summary of the performance of NS1 diagnostic kits is shown in Table 2, and they vary widely in sensitivity and specificity.

**Table 2.** Summary of commercially available dengue NS1 kit diagnostic performance

Kit	Type of test	Sensitivity (%)	Specificity (%)	Reference
SD BIOLINE Dengue Duo Kit (Standard Diagnostics, Suwon, Korea), catalogue number: 11FK45	RDT	87	87	^40^
Dengue NS1 Detect Rapid Test (InBios International, Seattle, WA, USA), catalogue number: DNS1-RD	RDT	77	97	^41^
Panbio Dengue Early ELISA (Abbott, Macquarie Park, NSW, Australia), catalogue number: 01PE40	ELISA	86	95	^41^
DENV Detect NS1 ELISA (InBios International, Seattle, WA, USA), catalogue number: DNS1-1	ELISA	96	100	^41^

Combining IgM and NS1 detection into one test, such as the Dengue Duo kit (Standard Diagnostics, Suwon, Korea), allows for high sensitivity during both the early (NS1) and late (IgM) phases of the disease and hence an improvement in overall test performance.^42^ This kit is also stable during prolonged storage at high temperature^43^ and has shown utility during outbreaks in resource-limited settings, albeit with an overall accuracy of <80%.^44^ Moreover, it has been shown to be an appropriate method of transporting and extracting RNA for serotyping at higher-level facilities, with up to 90% agreement with results from neat serum samples. Using it in this way could facilitate molecular epidemiology from rural areas.^45^

In secondary infections, the formation of antigen–antibody complexes with pre-existing IgG shortens the NS1 window of detection^21^ and reduces sensitivity.^46^ This drawback is concerning, as secondary infections tend to be more severe and their detection is important for surveillance and outbreak response.^47^

Another issue is that test sensitivity varies by serotype,^41,44,48^ meaning the introduction of new serotypes could be missed and the appropriate public health response delayed. For example, a retrospective analysis in Brazil found that 58/119 (49%) of NS1-negative samples were positive for DENV4. This led to an underreporting of cases and a delay in detecting the DENV4 outbreak.^48^ Overall, the strength of NS1 detection is in allowing rapid diagnosis during acute viraemia to guide clinical management, particularly when antibody serology is confounded by vaccination or previous flavivirus exposure in endemic areas, although knowledge of its sensitivity to circulating serotypes will be necessary to guide interpretation.

## Nucleic acid testing

Reverse transcription polymerase chain reaction (RT-PCR) is capable of detecting viral RNA with high sensitivity and specificity, providing relatively fast results (unlike virus isolation) and using only a single sample (unlike IgM/IgG serology). However, there are still significant barriers to using RT-PCR as a diagnostic test: it has a short window of opportunity (coinciding only with the viraemic window), has a high risk of contamination and requires the use of expensive laboratory equipment.^15,21^

Various techniques for performing RT-PCR using different primers and detection methods have been developed. These vary in sensitivity from 50% to 99%, but retain a high specificity (99–100%).^15^ Recently, real-time RT-PCR techniques have been developed that are faster to perform and carry less risk of contamination compared with traditional nested RT-PCR methods while retaining high sensitivities (80–100%) and specificities (99–100%).^49^

In order to address the prohibitive cost of RT-PCR, a variety of low-cost alternatives, such as hydrolysis-based assays, are being developed.^50^ These require only a heating block/water bath capable of maintaining a constant temperature rather than an expensive thermocycler. They have shown comparable sensitivity and specificity to conventional RT-PCR techniques as well as little or no cross-reactivity with related flaviviruses and therefore have the potential to improve dengue diagnostics in low-resource settings. Indeed, the POCKIT nucleic acid analyser (GeneReach Biotech, Taichung, Taiwan; GeneReach USA, Lexington, MA, USA) has recently been validated as a point-of-need PCR assay that is accurate and suitable for field deployment, although it cannot be used to serotype the virus.^50,51^ Another alternative would be thermocyclers that can operate with low power consumption and without a requirement for continuous electricity.^52^

While there is increased application of PCR for dengue diagnosis, a lack of standardisation in PCR methods makes it difficult to compare results across laboratories. In 2009, an external quality assessment of RT-PCR in 37 laboratories across 27 countries was carried out.^53^ From the 46 datasets examined (some laboratories provided more than one), only 9 were classified as acceptable for diagnostic purposes (capable of detecting true positives at >10^3^ genome equivalents/ml and with no false positives) and only 5 were acceptable for surveillance purposes (also capable of accurate serotyping). While most participating laboratories were based in Europe, where dengue is not endemic, this study nonetheless highlights the issues with a lack of standardisation. Many laboratories used ‘in-house’ methods and there was high heterogeneity in results even among those using the same techniques/primers. Given PCR’s current position as the most practical gold standard for surveillance and evaluation of new diagnostics, this variation is a concerning barrier for improving dengue diagnostics.

### Importance of serotyping

In addition to their diagnostic specificity, RT-PCR methods can be used to serotype the virus. Given the evidence of ‘displacement’ of the dominant circulating serotypes across time, serotyping may play an important role in predicting future outbreaks.^54,55^ From an international perspective, accurate serotyping will allow a better understanding of travelling waves in dengue fever transmission^56^ by identifying related outbreaks across borders. In addition, serotyping data can inform research into the multi-annual cross-country periodicity of dengue, thought to be related to the cycling of host immunity to different serotypes.^57^ Identifying related outbreaks can lead to improved international targeting of risk mitigation efforts.

Finally, vaccine efficacy has been shown to vary by serotype.^58^ Using nucleic acid data in post-vaccination follow-up can help identify viral genotype variants contributing to reduced vaccine efficacy and their geographical distribution, allowing public health officials to target vaccination programmes to areas with maximum efficacy and allowing vaccine development to continue in earnest. Overall, if standardised, PCR may allow accurate serotyping at central laboratories for triggering outbreak response, guiding vaccine development/implementation and providing data for transmission models, as well as providing relatively fast and accurate diagnosis for clinical management.

## Influence of dengue diagnostics on surveillance and control

The variable accuracy of diagnostic confirmation methods, coupled with their frequent lack of use, has key implications for surveillance studies. With the reliance on clinical diagnosis and physician motivation for reporting, it may be that dengue presenting without typical signs and symptoms or outside the dengue season may be misdiagnosed as a different febrile illness. In non-endemic countries, there is a low index of suspicion and a dengue diagnosis is likely to be missed clinically, as indicated by the high level of underreporting in an Angolan case-cluster study.^59^ This may explain the small number of reported cases in Africa despite the suitability of the climate for dengue transmission.^4^ Indeed, one study looking at the seroprevalence of dengue IgG antibodies in febrile outpatients of all ages (median 40.7 y) in Sudan found that 302/449 (67.3%) sera were positive, indicating a high rate of dengue infection that goes unrecognized. Many sera were also broadly neutralising, suggesting recurrent infections with different serotypes.^60^ Improving access to and the affordability of diagnostics is necessary for surveillance in regions like Africa.

While improvements in diagnostic tests and practices for dengue are essential, the imperfection of the ‘gold standard’ used in their evaluation underestimates their true accuracy. Latent class Bayesian modelling can be used to take this into account.^61^ Bayesian approaches formally acknowledge the fallibility of the gold standard and the variability of diagnostic test characteristics, allowing a better estimation of true prevalence.^62^ When this approach was applied to the Bangkok Armed Forces Research Institute of Medical Sciences reference IgM ELISA, a commonly used gold standard in diagnostic evaluation studies, dengue prevalence estimates rose from 15.3% to 24.3% and the sensitivity of the assay (previously assumed to be 100%) was calculated as 62%.^63^

Reported cases also need to be stratified by a confirmatory method to account for the different level of certainty associated with each method, improving model accuracy. Acknowledging and publicising the limitations in dengue figures by mandating stratified reporting to the WHO could incentivise improvements in reporting practices and roll-out of diagnostic tests, thus improving surveillance. Highlighting the regions and times that show the greatest uncertainty, as well as the causes of that uncertainty, would help in devising policy interventions to improve reporting. Finally, open acknowledgement of these uncertainties in reported figures will help both researchers and policymakers account for possible bias and thus better inform their practice. By coupling proactive quality improvement in reporting and diagnostics, increased compliance with current anti-dengue control measures and the roll-out of the newly licensed vaccine, dengue may be brought under control from a public health perspective.

## Conclusions

Most cases of dengue are diagnosed based on signs and symptoms alone, creating substantial uncertainty due to the non-specific and non-uniform case definitions used. Even when diagnostic methods are used, each method reviewed has wide variations in accuracy depending on the methodology/kit and epidemiological context. This carries significant consequences, as diagnostic strategy needs to be tailored to regional situations. In this review we outline the situations where each diagnostic test would be most useful epidemiologically based on its strengths and limitations, as well as some of the implications of recent developments in diagnostics. Finally, having highlighted the huge variability in accuracy, we caution against treating all reported dengue cases as equal and call for stratified reporting by diagnostic method, without which accurately accounting for uncertainties in model development and public health planning becomes nearly impossible.

## Notes

Present address: Oxford University Medical School, John Radcliffe Hospital, Oxford, OX3 9DU, UK.
